# Supplementary material for: Sulforaphane Inhibits the Expression of Long Noncoding RNA H19 and Its Target APOBEC3G and Thereby Pancreatic Cancer Progression
Source: Cancers (Basel). 2021 Feb 16;13(4):827. doi: 10.3390/cancers13040827 (PMC7920255; doi:10.3390/cancers13040827)
Supplement: Supplementary file 1 [file cancers-13-00827-s001.pdf]

cancers

MDPI

*Supplementary Materials*

# Sulforaphane Inhibits the Expression of Long Noncoding RNA H19 and its Target APOBEC3G and Thereby Pancreatic Cancer Progression

**Yiqiao Luo, Bin Yan, Li Liu, Libo Yin, Huihui Ji, Xuefeng An, Jury Gladkich, Zhimin Qi, Carolina De La Torre and Ingrid Herr ***

**Table S1.** Primer sequences.

| | Forward | Reverse |
|---|---|---|
| H19 | GCACCTTGGACATCTGGAGT | TTCTTTCCAGCCCTAGCTCA |
| MALAT | CTCCCCACAAGCAACTTCTC | TTCAACCCACCAAAGACCTC |
| HOTAIR | GGTAGAAAAAGCAACCACGAAGC | ACATAAACCTCTGTCTGTGAGTGCC |
| HOTTIP | CCTAAAGCCACGCTTCTTTG | TGCAGGCTGGAGATCCTACT |
| PVT1 | GCCCCTTCTATGGGAATCACTA | GGGGCAGAGATGAAATCGTAAT |
| A3G | GGGACCCAGATTACCAGGAG | GCAGATTATTCCAAGGCTCAA |
| β-actin | AATCGTGCGTGACATTAAGGAG | ACTGTGTTGGCGTACAGGTCTT |

**Table S2.** Identification of 17 candidate genes.

| Gene Symbol | Gene Name | Tumor type | Function |
|---|---|---|---|
| OSGIN1 | Oxidative stress induced growth inhibitor | Breast cancer, Hepatocellular carcinoma | Tumor suppressor |
| TNFSF9 | TNF superfamily member 9 | Hepatocellular carcinoma | Tumor suppressor |
| ADI1 | Acireductone dioxygenase 1 | Hepatoma, Prostate cancer | Tumor suppressor |
| TRIM16 | Tripartite motif containing 16 | Breast cancer, Ovarian cancer, Prostate Cancer | Tumor suppressor |
| TMEM127 | Transmembrane protein 127 | Pheochromocytomas, Renal cancer | Tumor suppressor |
| **APOBEC3G** | **Apolipoprotein B MRNA Editing Enzyme Catalytic Subunit 3G** | **Pancreatic cancer, Mesenchymal gliomas** | **Tumor promotor, associated with immunity** |
| ASAH1 | N-Acylsphingosine Amidohydrolase 1 | Breast cancer | Tumor promotor |
| TARS | Threonyl-tRNA synthetase | Breast cancer, Pancreatic cancer, Ovarian cancer | Tumor promotor |
| BIRC3 | Baculoviral IAP repeat containing 3 | Pancreatic cancer, Breast cancer, Colorectal cancer | Tumor promotor |
| APOBEC3B | Apolipoprotein B MRNA Editing Enzyme Catalytic Subunit 3B | Breast cancer, Hepatocellular carcinoma | Tumor promotor |
| RAPB3IP | RAB3A interacting protein | Esophageal squamous cell carcinoma | Tumor promotor |
| MYO1B | Myosin IB | Cervical cancer, Prostate cancer | Tumor promotor |
| TCF7L2 | Transcription factor 7 like 2 | Cervical cancer, Gastric cancer, Breast cancer | Tumor promotor |
| DTX3L | Deltex E3 ubiquitin ligase 3L | Melanoma, Glioma | Tumor promotor |
| WARS | Tryptophanyl-tRNA synthetase | Oral cancer, Gastric cancer | Tumor promotor |
| CLU | Clusterin | Prostate cancer, Colorectal cancer | Tumor promotor |
| CDKN2AIP | CDKN2A interacting protein | Ovarian cancer, Lung cancer | Tumor promotor |

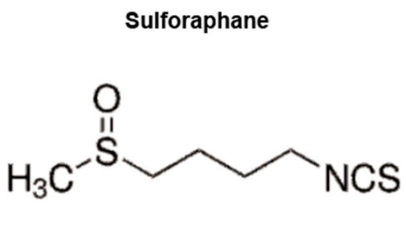


**Figure S1.** Chemical structure of sulforaphane.

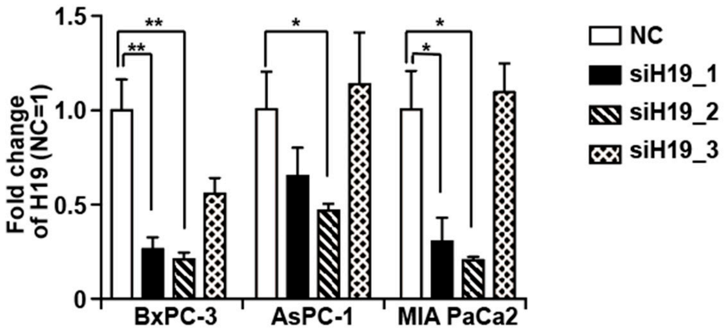


**Figure S2.** Identification of siH19_2 as the most efficient lncRNA H19 inhibitor. (**A**) The three different siRNA constructs targeting H19, namely, siH19_1, siH19_2 and siH19_3, along with a nonsense siRNA control (NC), were lipo-transfected into BxPc-3, AsPC-1 and MIA-PaCa2 cells at 20 µg each. Twenty-four hours later, the expression of H19 was detected by RT-qPCR. The fold change of H19 expression in the knockdown group was normalized to that in the nonsense siRNA control group, which was set to 1. * $p < 0.05$, ** $p < 0.01$.

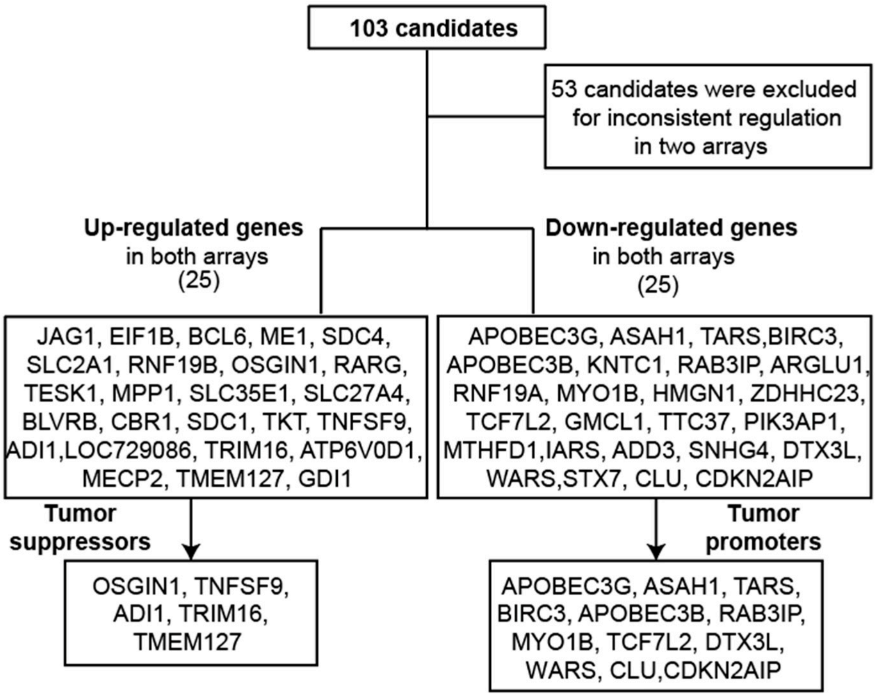

**Figure S3.** *In* silico identification of 17 H19-related candidate genes. A total of 103 candidates were selected according to the threshold of adjusted *p* value < 0.01 in two gene arrays as described in Fig. 3. Among them, 53 candidates were excluded because of inconsistent expression trends within the two gene arrays. Among the resulting 50 candidates, 25 genes were upregulated and 25 genes were downregulated in both gene arrays. By the use of the PubMed and Web of Science databases and the key words "cancer promoter" or "cancer suppressor", 17 candidates were selected, including 5 tumor suppressors and 12 tumor promoters.

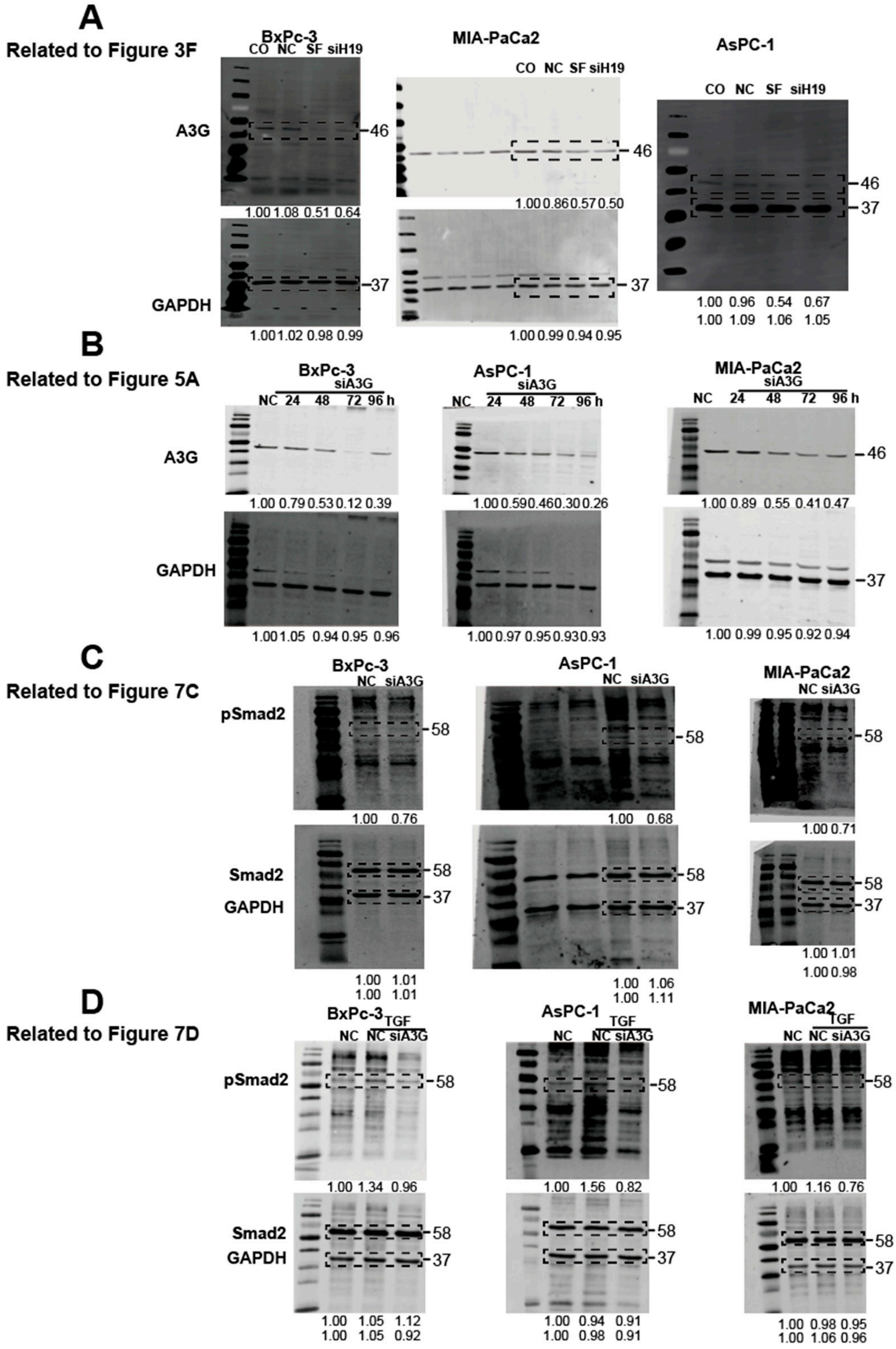


Luo et al., Suppl. Fig. S4

**Figure S4.** Original, crude Western blot images with molecular weight markers. (**A**)Related to Fig. 3F. (**B**) Related to Fig. 5A. (**C**) Related to Fig. 7C. (**D**) Related to Fig. 7D.

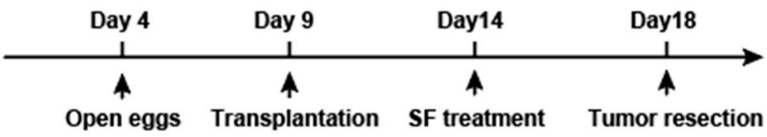


**Figure S5.** Treatment scheme. After cleaning, the eggs were incubated to start the development of the chick embryo, which is scheduled at day 1 at the first day of incubation. At day 3 of development, a hole was cut into the egg shell, to open it for xenotransplantation. At the developmental day 9, $10^6$ MIA-PaCa2 cells in 50 µl Matrigel™, were transplanted onto the scratched CAM. At day 14, 100 µl of a 100 µM sulforaphane solution, or 100 µl saline, was injected directly into the CAM vessels that supplied the xenograft tumors. At 18 day of chick development, all embryos were humanely euthanized.

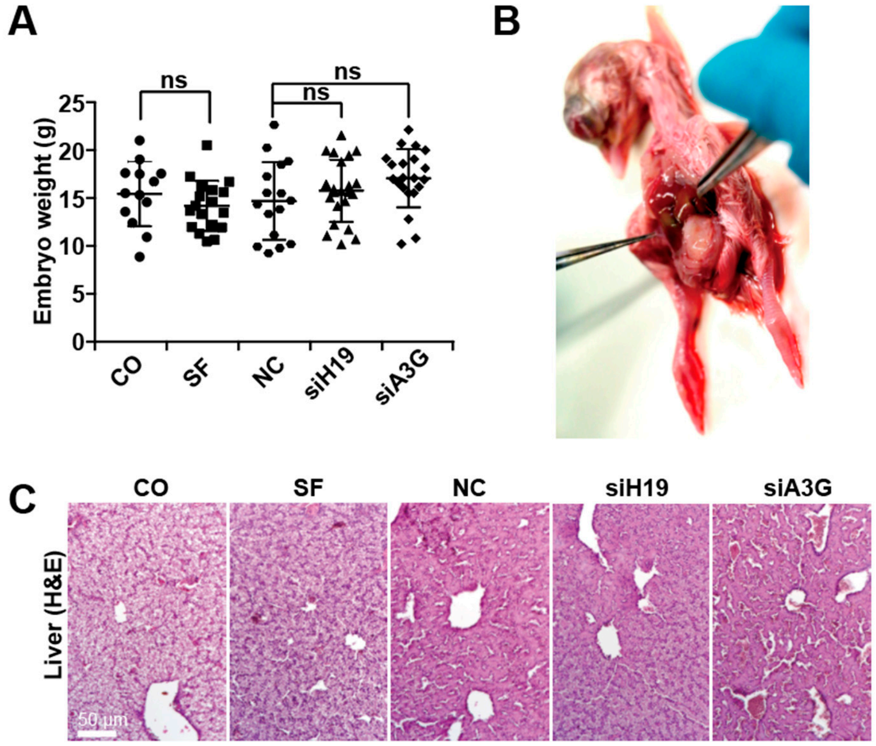


**Figure S6.** siH19, siA3G or sulforaphane treatment did not induce side effects in vivo. (**A**) After resection of xenograft tumors as described in Fig. 6, the weight of each chick embryo was determined and is presented as a black dot, and the mean weights per group are given. (**B**) Representative image of a chick embryo at day 18 of development and liver resection. (**C**) Representative H&E staining of frozen liver tissue sections from each group.
